# Research on Wind Environment Characteristics of the QiTai Radio Telescope Site Based on Wind Tower Measurements

**DOI:** 10.3390/s26010051

**Published:** 2025-12-20

**Authors:** Feilong He, Laibing Li, Qian Xu, Na Wang, Shijiao Zhang, Hui Wang, Guljaina Kazezkhan, Xiaoman Cao

**Affiliations:** 1 State Key Laboratory of Radio Astronomy and Technology, Xinjiang Astronomical Observatory, Chinese Academy of Sciences, Urumqi 830011, China; hefeilong@xao.ac.cn (F.H.); zhangshijiao@xao.ac.cn (S.Z.); wanghui@xao.ac.cn (H.W.); jaina@xao.ac.cn (G.K.); caoxiaoman@xao.ac.cn (X.C.); 2 School of Mechanical Engineering, Xinjiang University, Urumqi 830047, China; 3 Xinjiang Key Laboratory of Radio Astrophysics, Urumqi 830011, China; 4 University of Chinese Academy of Sciences, Beijing 100049, China

**Keywords:** radio telescope site, wind sensor, wind environment measurement, data processing, wind field characteristics

## Abstract

Wind disturbance is one of the key factors affecting the high-precision pointing of large-aperture radio telescopes. Therefore, it is indispensable to monitor the wind environment of the site. This enables the acquisition of wind environment data, facilitating targeted wind-resistant design to maintain the observational performance of the radio telescope. A 60 m high wind tower is located within the QTT (QiTai Radio Telescope, 110 m) site. This study investigates the wind environment characteristics based on the wind data for the entire year of 2021. The analysis of anomalous data from the wind tower indicates that these are mainly caused by local freezing rain and snow conditions. The temporal variations and vertical distribution characteristics of the wind environment were analyzed. On an annual basis, winds predominantly originate from north–south, while those from east–west are relatively less frequent; 90% of the winds are less than 4 m/s; the maximum recorded wind speed is 22.29 m/s; the prevailing winds are from the SSE (south-southeast) direction. On a monthly basis, the distributions of wind direction and speed exhibit a distinct seasonal cycle, with wind speeds being relatively lower in winter. On a diurnal basis, the wind direction undergoes a reversal, with northerly winds prevailing during the day and southerly winds at night; the diurnal wind speed distribution shows that nocturnal wind speeds are relatively stable and lower. Daily wind speed statistics indicate that there were 79 days on which 90% of wind speeds throughout the day were less than or equal to 2 m/s. Compared to sites of other telescopes of a similar class, the wind environment at the QTT site is relatively favorable.

## 1. Introduction

The QiTai Radio Telescope (QTT), which is currently under construction, is fully steerable. It has an aperture of 110 m, an operating frequency band of 150 MHz to 115 GHz, and a maximum repeat pointing accuracy of 2.5″ [1]. For a large radio telescope, its excellent performance relies not only on its own structural design, manufacturing processes, and assembly precision, but also on the site environment [2,3,4]. Nan et al. [5,6] conducted field investigations on the topography, climate, and engineering environment of karst depressions in Guizhou Province for more than 12 years and finally identified the site for the Five-hundred-meter Aperture Spherical radio Telescope (FAST). The National Astronomical Observatory of Japan (NAOJ) plans to build a 50 m class single-dish submillimeter radio telescope and has statistically analyzed meteorological data for candidate sites to evaluate the wind environment [7]. Observational data from the Green Bank Telescope (GBT) indicate that the currently observable frequency bands are entirely constrained by the wind speed conditions at the site [8].

**Figure 1:**
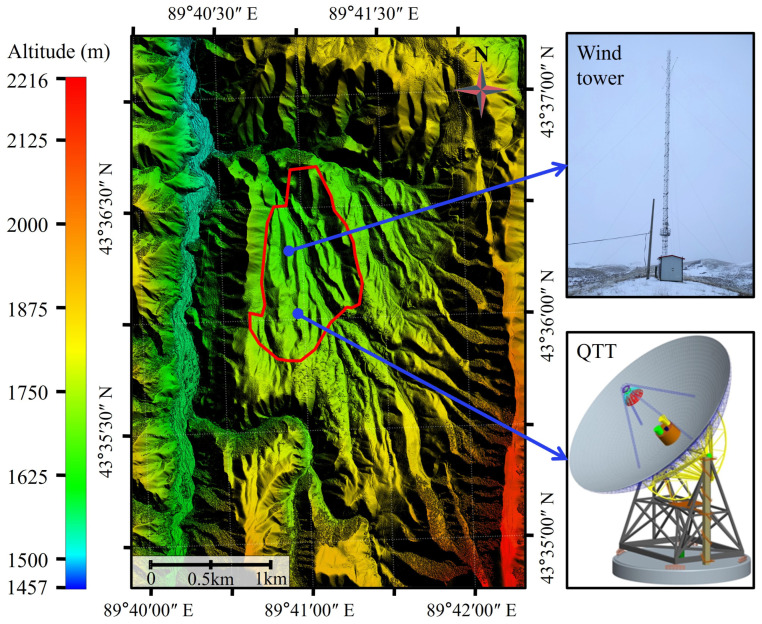
The QTT site. The red line indicates the site area.

QTT is located in the northern foothills of the eastern Tianshan Mountains, with coordinates 43°36′4.03″ N and 89°40′56.99″ E, and an elevation of approximately 1760 m. It is a rectangular basin, enclosed by mountains on all sides, whose ridges range in height from 1820 to 2250 m (as shown in Figure 1). This type of terrain not only provides shielding from electromagnetic interference but also effectively blocks some wind inflow. However, wind blowing toward the antenna remains a significant influencing factor. As the telescope aperture increases and the observing frequency rises, the influence of wind disturbances on telescope pointing accuracy becomes increasingly significant [9].

To improve the telescope’s robust observation duration and data quality in windy environments, antenna design for wind disturbance mitigation is indispensable. Jiang et al. [10] designed a Fractional-Order Active Disturbance Rejection Controller (FOADRC) to enhance the pointing accuracy of the antenna under wind disturbance. The simulation results show that FOADRC has better resistance to wind disturbances and faster convergence speed than other industrial controllers. Wang et al. [11] established a parametric finite element model of a radio telescope to deal with the wind load that affects the signal reception accuracy. They explored the relationship between the size parameters of the structural components and the dynamic response to wind load, and finally optimized the structural size parameters of each component of the telescope. Li et al. [12] obtained the wind load distribution and wind coefficients of the antenna reflector surface under different windward attitudes by numerical simulation, and constructed a proxy model for calculating the wind coefficients, aiming to provide rapid wind load information to support the anti-wind disturbance design. The aforementioned studies indicate that effective anti-wind disturbance design is a crucial pathway to enhancing telescope observational performance. However, its successful implementation is highly dependent on an accurate understanding of the wind environment characteristics at the site.

Ries et al. [13] found that at wind speeds above 4 m/s, wind-induced feed-arm deflections become the dominant constraint on the high-frequency performance of the GBT. They implemented an optical quadrant detector to provide a correction signal for these wind-induced pointing errors. Hashimoto et al. [14] measured the wind speed of the site and the vibration acceleration of the main reflecting surface of the antenna for the Nobeyama 45 m radio telescope. They found that the antenna’s vibration pattern depends on the direction of the wind load, and the pointing error will be triggered when the wind speed is greater than 5 m/s. Wang et al. [15] installed an automatic weather station to obtain first-hand wind data at the Rikaze radio telescope site and analyzed the wind data for the past year to summarize the current situation of the wind environment. In summary, detailed research and assessment of the site’s wind environment characteristics are essential tasks for ensuring the telescope’s observational performance.

Previous research on the QTT wind environment has largely focused on preliminary surveys and engineering mitigation strategies. The basic wind environment of the site was investigated [1]. Subsequent efforts concentrated on wind field reconstruction using CFD simulations [16] and wind disturbance mitigation technologies, including wind speed forecasting [17], servo control optimization [18], and wind field regulation via fences [19]. These studies primarily utilized wind data as a boundary condition or validation metric for engineering models.

However, a comprehensive understanding of the natural wind field’s intrinsic characteristics remains limited. This study investigates the wind field characteristics at the site in greater detail by analyzing a full year of multi-level measured data to: (1) Investigate the causes of anomalous data to improve wind data reliability; (2) Reveal the physical mechanisms driving the site’s unique wind distribution; (3) Provide direct information support for wind-resistant design and future observation scheduling of the QTT.

## 2. Measurement System and Data Preprocessing

### 2.1. Wind Tower and Sensor Setup

**Figure 2:**
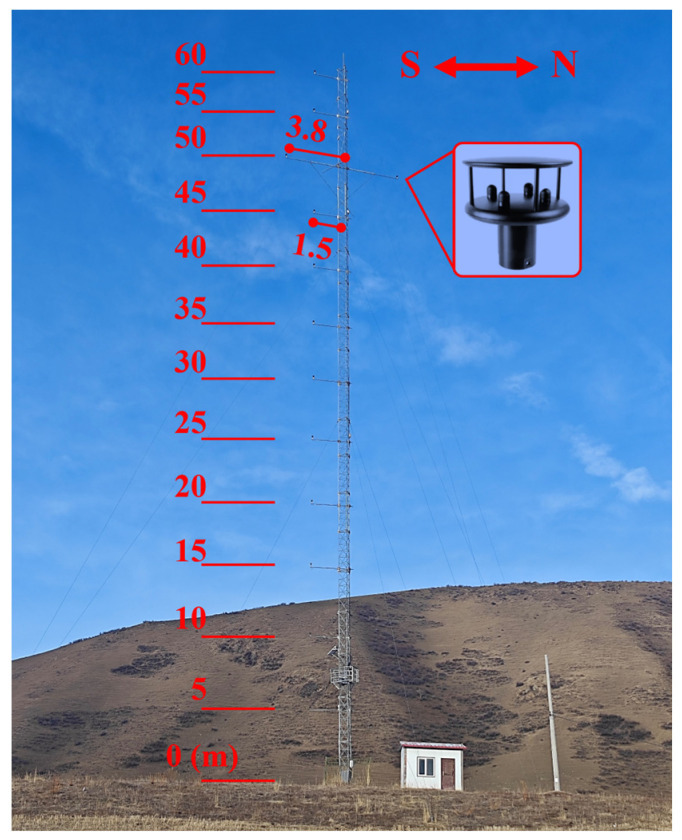
Configuration of the 60 m wind tower.

The wind monitoring system is installed on a 60 m tower located within the QTT site. To capture the vertical wind profile, the tower is equipped with 12 measurement levels spaced at 5 m intervals, ranging from 5 m to 60 m above ground level (as shown in Figure 2). At heights of 5, 10, 15, 20, 25, 30, 35, 40, 45, 55, and 60 m, a single sensor is mounted on a 1.5 m boom oriented toward the South. To evaluate flow distortion and ensure accuracy, the 50 m level is equipped with two sensors on longer 3.8 m booms (facing South, North). This geometry helps minimize tower shadowing effects for the prevailing North–South wind directions. The wind speed and direction are measured using WindSonic 2D ultrasonic anemometers (Gill Instruments, Limington, Hampshire, UK). Unlike mechanical cup anemometers, these solid-state sensors utilize ultrasonic pulse time-of-flight technology, which minimizes inertia-induced errors and eliminates moving parts prone to mechanical failure in harsh environments. The sensors have a measurement range of 0–60 m/s with a resolution of 0.01 m/s and a direction accuracy of ±3°. All sensors are calibrated by the factory before leaving the factory. The parameters of these ultrasonic anemometers are shown in Table 1.

### 2.2. Data Acquisition Strategy

Data logging is performed using a Campbell Scientific CR3000 Micrologger, a rugged data acquisition system designed for operation in temperatures ranging from −25 °C to +50 °C. All the collected data are provided with a time series by the same clock module. The clock module regularly synchronizes with the standard time via the network. The sampling frequency is set to 0.1 Hz (one sample every 10 s), with each sample consisting of a 1-s signal integration. The logger processes these raw samples to generate 1-min statistical data, which includes: sampling wind speed and direction, maximum wind speed and its corresponding direction, and average wind speed. “Sampling wind speed and direction” refers to the wind speed and direction data from the first sample of each minute. “Maximum wind speed and its corresponding direction” refers to the highest wind speed recorded among the six samples taken each minute, along with its corresponding direction. “Average wind speed” is the average of the six wind speed samples collected each minute.

### 2.3. Performance Analysis and Quality Control

For the analysis of the site’s wind environment characteristics, this paper uses wind observation data from the 15 m, 25 m, 35 m, 45 m, and 55 m layers of the wind tower (the sensor at the 60 m layer was damaged). The wind observation data were collected from 1 January to 31 December 2021. The wind tower collected a total of 523,033 pieces of data, at a frequency of one record per minute. The anomalous data were statistically classified into two main types: typical anomalous data and other anomalous data. Typical anomalous data refers to data marked with identifiers such as “NAN,” “999,” and “1000.” Other anomalies include instances of markedly anomalous values, missing data, and time duplicates. Markedly anomalous values are defined as recorded values that exceed the preceding value by a factor of more than five; missing data refers to data that was not recorded; and time duplication denotes instances where two data records occur within the same minute. Among these, there were 7581 typical anomalous data, accounting for 1.45% of the total, and 2592 other anomalous data, accounting for 0.50%. Statistics for the anomalous data are shown in Table 2.

**Figure 3:**
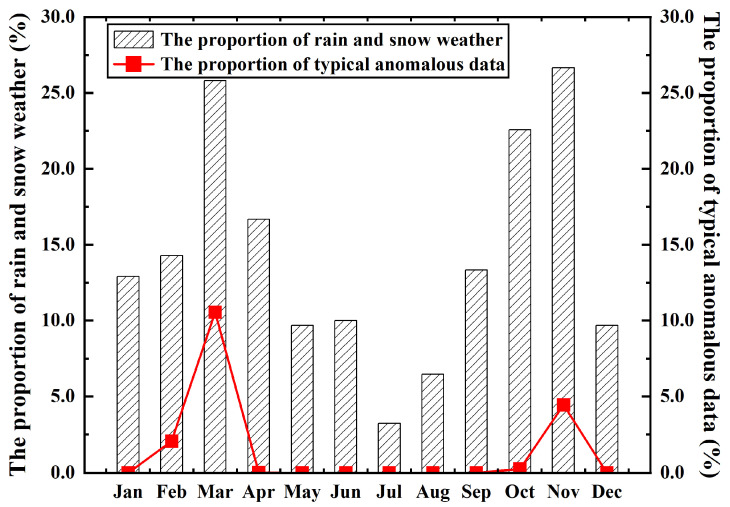
Correlation of typical anomalous data with rain and snow weather.

As seen in Table 2, typical anomalous data occurred in six months of the year. The frequency of such data was notably high in March and November, accounting for 10.55% and 4.45% of each month’s wind data, respectively. In terms of timing, these occurrences were relatively concentrated and exhibited low randomness. The analysis suggests that the generation of typical anomalous data is attributed to rain and snow weather conditions at those times. A statistical analysis of year-round rain and snow weather in Qitai County was conducted, along with a correlation analysis, as shown in Figure 3. The figure indicates that March and November have a higher frequency of rain and snow, which shows a strong correlation with the occurrence of typical anomalous data. Although other months also experience rain and snow, the correlation is weaker. This is attributed to the fact that November and March are the onset of winter and early spring, respectively. During these periods, the meteorological environment is characterized by low temperatures and high humidity. Under such conditions, sensor surfaces are prone to icing. This, in turn, leads to malfunctions in the wind observation equipment. The problem of missing wind data due to sensor icing has garnered considerable attention in meteorological observation, with numerous researchers having conducted studies on the anti-icing capabilities of wind sensors [20,21]. Consequently, in the subsequent selection of wind observation equipment, enhanced consideration must be given to the anti-icing performance of wind sensors. Among other types of anomalous data, missing data accounts for a substantial proportion, potentially attributable to power outages or instrument downtime for maintenance. Furthermore, wind observation equipment, when influenced by the meteorological environment, may also generate sporadic instances of other anomalous data.

Due to the difficulty in repairing long continuous segments of anomalous data through interpolation, such segments are consequently removed. Furthermore, intervals exhibiting frequent intermittent occurrences of anomalous data within a given period are also excluded. Even if these intervals contain seemingly normal data points, such data are deemed unreliable and are therefore discarded along with the anomalous data. For sporadic anomalous data occurring within a period, interpolation methods are primarily employed for repair. In instances of data with duplicate timestamps, only one of the two data points recorded per minute is retained, typically after an assessment involving adjacent data values.

## 3. Analysis of Wind Environment Characteristics at the Site

### 3.1. Analysis of Annual Wind Environment Characteristics

**Figure 4:**
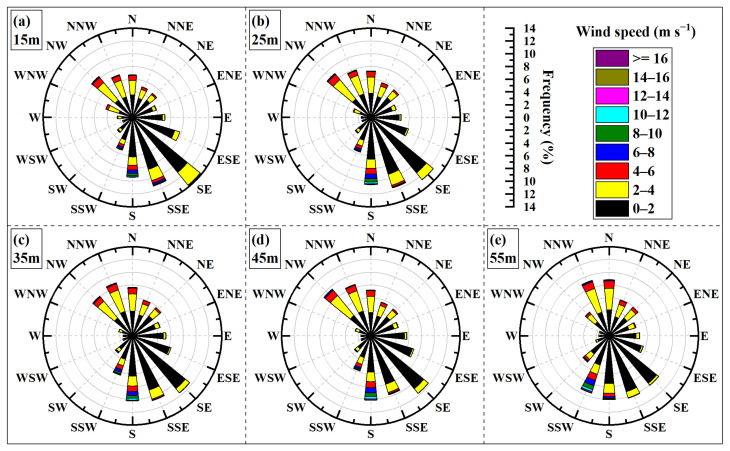
Sampling wind rose diagram throughout the year.

**Figure 5:**
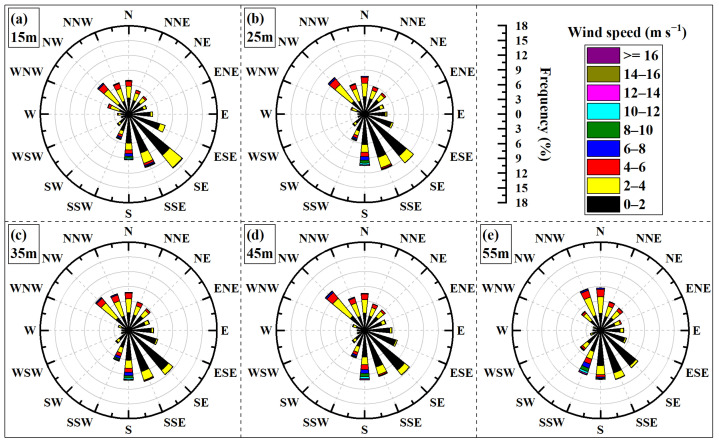
Maximum wind rose diagram throughout the year.

The wind rose diagram is a graphical tool used to depict the statistical distribution of wind speed and direction at a specific location over a period. It is so named due to its characteristic shape resembling a rose flower. In a wind rose diagram, concentric circles radiating from the center represent frequency scales, while different color bands within each directional sector indicate various wind speed ranges. The wind rose diagrams presented in this paper divide the wind directions into 16 standard wind directions, with each wind direction corresponding to a sector. The names, symbols, and corresponding angular ranges for these 16 standard wind directions are detailed in Table 3. Wind rose diagrams were generated based on sampling wind data and maximum wind data, respectively, as illustrated in Figure 4 and Figure 5. As observed from these figures, the prevailing wind directions at the site are predominantly from the south and north (cardinal directions: north, south, east, west). For purpose of subsequent analysis, Description of wind directions (16 standard wind directions) are classified as follows: NW, NNW, N, NNE, and NE are categorized as north; SE, SSE, S, SSW, and SW are categorized as south; ENE, E, and ESE are categorized as east; and WNW, W, and WSW are categorized as west.

Figure 4 shows sampling wind rose diagrams for five different height layers. In these diagrams, the prevailing wind directions are approximately aligned along a “diagonal” axis between NW-SE, with wind frequencies from other directions gradually decreasing on either side of this dominant axis. The variation in wind speed and direction with height shows minimal change in the east–west, whereas it is more pronounced in the north–south. The prevailing winds in the north gradually shift from the NW towards N with increasing height, while the prevailing winds in the north shift from the SE towards SSW. Regarding wind speed distribution, winds are predominantly below 4 m/s (represented by black and yellow color bands). As height increases, the direction associated with relatively higher wind speeds transitions from S towards SSW.

The comprehensive analysis suggests that the local terrain changed the distribution of wind flow in space. For example, upstream there are no mountains with vertical wind directions in the south (see Figure 1), which has a weak blocking effect on the wind from all directions of south. The frequency of south winds is relatively high, and their speed is also high. Due to the sheltering by mountains to its west, the prevailing wind direction at the lower levels of the wind tower is primarily characterized by winds flowing along the river valley that passes through the site. With increasing height, the sheltering effect of the western mountains on SSW winds diminishes, and the frequency of winds from the SSW direction becomes more prominent. Furthermore, the SSW direction from the wind tower aligns with a gap in the western mountains, which acts as a conduit for strong winds originating from snow-capped mountains. The characteristics of the regional wind field are influenced not only by large-scale atmospheric circulation and meso- and micro-scale thermal factors but also significantly by local topographical conditions [22,23]. Influenced by the mountainous terrain, various field meteorological stations in Qitai County have reported inconsistent prevailing wind directions [24].

**Figure 6:**
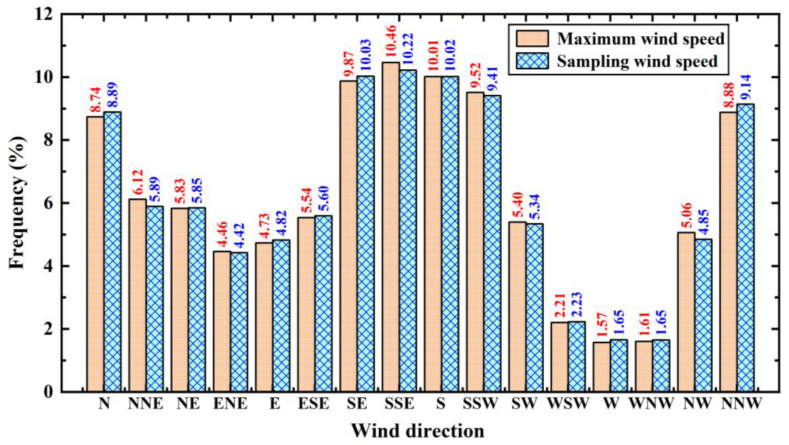
Frequency distribution of maximum, sampling wind speeds at 16 standard wind directions.

Figure 5 shows maximum wind rose diagrams for the same five height layers. Compared to Figure 4, the distribution patterns of maximum wind speed and its corresponding direction, as well as their variations with height, show considerable similarity to those of the sampling wind rose diagrams, with only minor discrepancies observed for certain height layers. Figure 6 shows a statistical comparison of wind frequencies for maximum wind speeds and sampling wind speeds at the 55 m height layer in the 16 standard wind directions. The figure clearly indicates that the frequency differences between these two datasets are minimal.

The average wind speed profile describes the variation in average wind speed with height above ground and can indicate the wind loads experienced by an antenna at different heights. This profile is commonly described using either the logarithmic law or the power law. In this study, the power law expression, which is considered more suitable for engineering calculations, is adopted to fit the average wind speed profile. The mathematical expression is as follows:

(1) $$\frac{v_{i}}{v_{0}}={\left(\frac{z_{i}}{z_{0}}\right)}^{\alpha }$$

In the formula: α is the average wind speed profile exponent; $z_{0}$ is the reference height; $z_{i}$ represents other specific heights; $v_{i}$ is the average wind speed at height $z_{i}$; $v_{0}$ is the average wind speed at the reference height $z_{0}$. In this paper, i=1,2,3,4; $z_{0}=15$ m, $z_{1}=25$ m, $z_{2}=35$ m, $z_{3}=45$ m, $z_{4}=55$ m.

Taking the logarithm of both sides of Formula (1), the following rearrangement is established:

(2) $$\mathrm{lg}(\frac{v_{i}}{v_{0}})=\alpha \mathrm{lg}(\frac{z_{i}}{z_{0}})$$

Let $y_{i}=\mathrm{lg}(v_{i}/v_{0})\;$ and $x_{i}=\mathrm{lg}(z_{i}/z_{0})$, transforming the relationship into the linear equation $y_{i}=\alpha x_{i}$. The value of α is found using the least squares method. When the sum of squared residuals, $\mathrm{RSS}=\sum_{i=1}^{n}{\left(y_{i}-\alpha x_{i}\right)}^{2}$, is minimized, the corresponding value of α is taken as the fitted average wind profile exponent.

**Figure 7:**
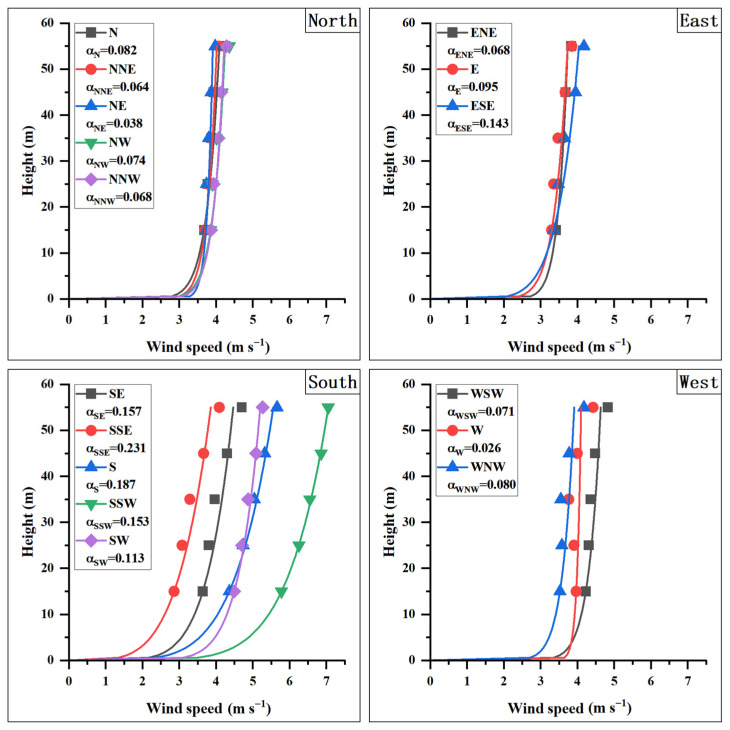
The average wind speed profile at 16 standard wind directions.

The higher the wind tower, the more representative the wind data collected by the top-layer sensor is of the regional wind characteristics. Using the sampling wind speed and direction at 55 m as the filtering criteria (with wind data from other heights grouped in accordance with the 55 m sampling data), the data samples were divided into 16 standard wind directions, and data for wind speeds exceeding 3 m/s were retained. Then, the sampling wind speed for each layer within each group was averaged to obtain the average wind speed at different heights and the fitted average wind speed profile, as shown in Figure 7. In the figure, the α value for most wind directions from the east, west, and north is less than 0.1, indicating that the difference in average wind speed at different heights is relatively small. However, for all southerly wind directions, the α value is greater than 0.1, meaning that the wind pressure difference experienced between the top and bottom of the future antenna will be relatively large.

**Figure 8:**
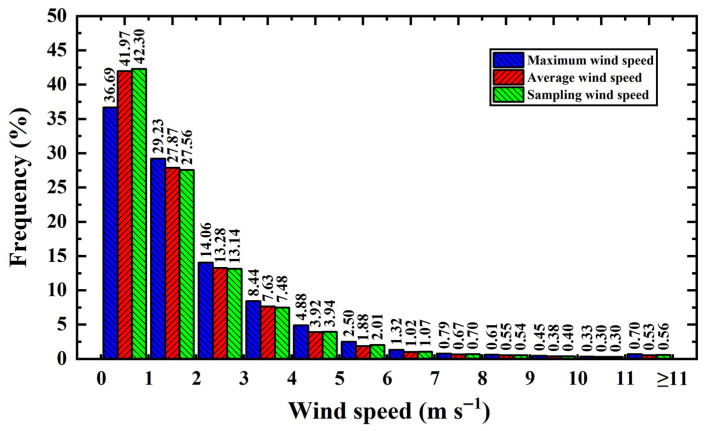
Frequency distribution of maximum, average, and sampling wind speeds.

Figure 8 shows the frequency distributions of maximum, average, and sampling wind speeds across different speed intervals at the 55 m height layer. Statistical analysis of wind frequencies at the QTT site shows that for sampling wind speeds, 94% of winds are below 5 m/s, and 99% are below 10 m/s. In comparison, at other large-aperture radio telescope sites, 90% of wind speeds at the Large Millimeter Telescope (LMT) site are below 10 m/s [25]. At the GBT site, 47% of average wind speeds are below 5 m/s, and 80% are below 10 m/s [26]. Compared to other sites, the QTT site exhibits relatively favorable wind environment conditions.

**Figure 9:**
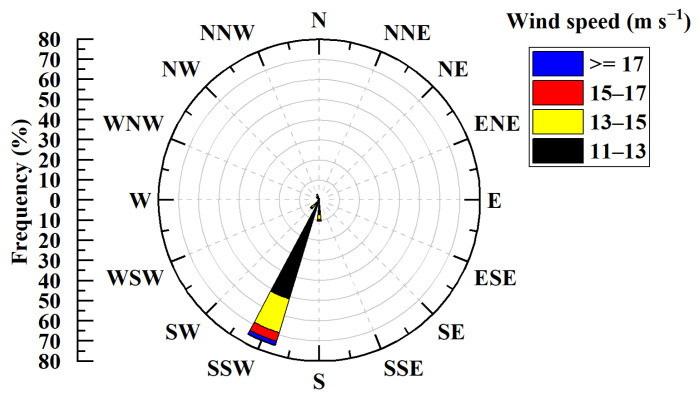
Wind rose diagram with a 55 m height layer, greater than 11 m/s wind speed.

Sampling wind data for wind speeds exceeding 11 m/s at the 55 m height layer were selected and plotted as a wind rose diagram in Figure 9. The figure shows that winds exceeding 11 m/s originate predominantly from the SSW, accounting for 75.43% of the frequency of such strong winds. This is followed by the S, with a frequency of 10.52%, which is approximately one-seventh of that from the SSW. Consequently, future preparedness and mitigation measures for strong winds should primarily focus on the SSW.

### 3.2. Analysis of Monthly Wind Environment Characteristics

**Figure 10:**
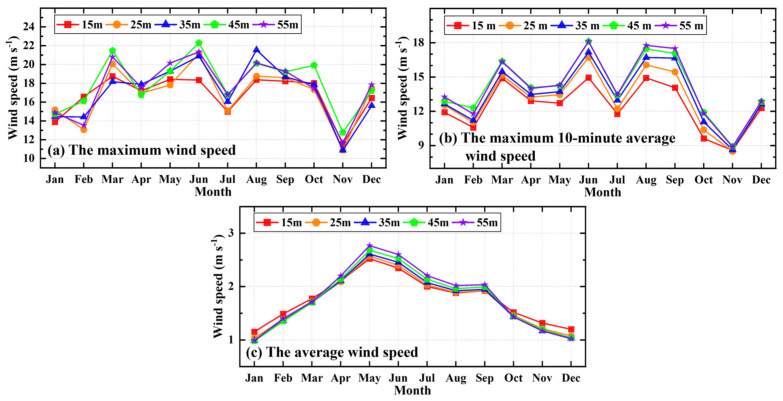
Monthly distribution of the maximum wind speed, the maximum 10-min average wind speed, and the average wind speed at different heights (15–55 m).

Figure 10 illustrates the monthly distribution of the maximum wind speed, the maximum 10-min average wind speed, and the average wind speed across different heights (15–55 m). As shown in the figure, the monthly variations in the maximum wind speed and the maximum 10-min average wind speed exhibit strong consistency and a distinct seasonal cycle. Peaks are observed in March, June, and August, with the highest values occurring in June (22.29 m/s) and the lowest in November (10.87 m/s). The monthly average wind speed presents a unimodal distribution, peaking in May and reaching minimums in winter (January and December). The observational data from different heights show a high degree of consistency in their monthly trends. In the vertical direction, the maximum wind speed does not show a clear regularity with height, whereas the maximum 10-min average wind speed generally increases with height. For the monthly average wind speed, a critical threshold is observed at approximately 1.8 m/s. When the wind speed exceeds this threshold, the speed increases with height; conversely, when the wind speed is below 1.8 m/s, the average wind speed decreases as height increases. According to the operational standards of the China Meteorological Administration, a “gale day” is defined as a day with an instantaneous wind speed exceeding 17.2 m/s (10 m above ground level). Based on statistics from the Qitai County meteorological department, the town of Banjiegou averaged only one gale day per year between 2015 and 2020, representing the lowest frequency among all towns in Qitai County [24].

**Figure 11:**
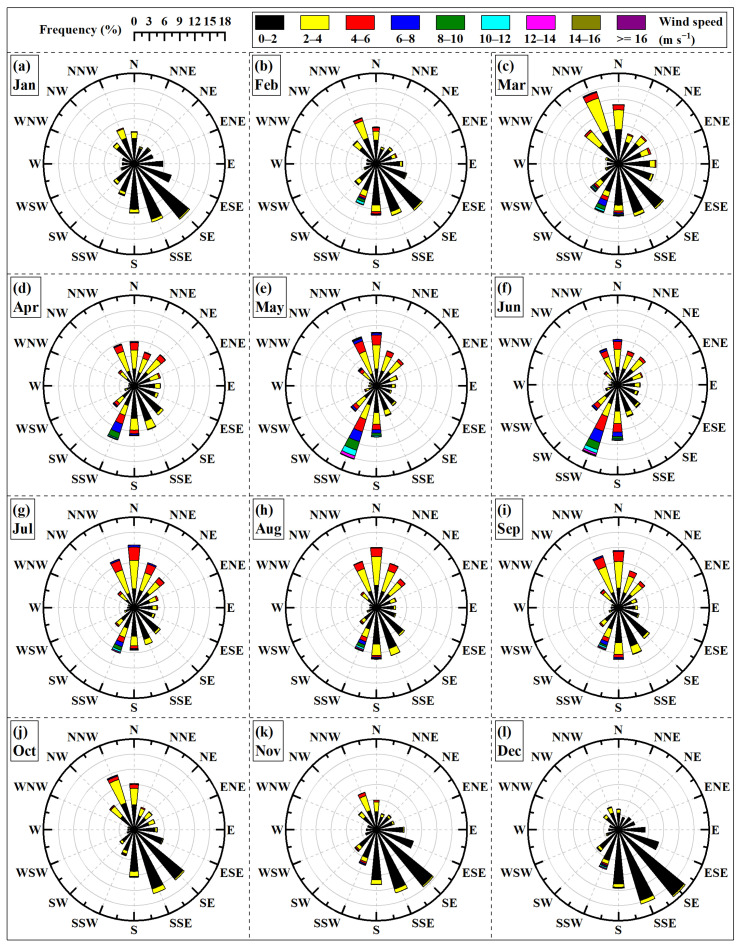
Sampling wind rose diagram from January to December.

Wind rose diagrams for the period from January to December were generated using sampling wind data from the 55 m height layer, as illustrated in Figure 11. Based on the meteorological definition of seasons, the months from March to May are defined as spring, June to August as summer, September to November as autumn, and December to February of the following year as winter. As observed in Figure 11, the prevailing wind directions exhibit a distinct seasonal cycle. In the south, from January to June, the prevailing wind direction veers from SE towards SSW, while the frequencies of winds from other directions vary in accordance with this shift. Subsequently, from July to December, the prevailing wind direction shifts back from SSW towards SE. In the north, from January to August, the prevailing wind direction veers from NNW towards N; then, from September to December, it shifts back from N towards NNW. The frequencies of winds from easterly and westerly directions vary in conjunction with these shifts in the prevailing north–south winds. Furthermore, seasonal variations are also observed in the distribution of wind speeds across different speed intervals. As shown in Figure 11, the frequency of winds exceeding 4 m/s increases steadily from January to May, then gradually decreases from June to August. It experiences a slight rise in September before decreasing steadily again from October to December. Of the four seasons, winter experiences relatively lower wind speeds. These findings are consistent with observations from the nearby Banjiegou and Daotiaoling meteorological stations [24].

Table 4 presents the monthly distribution of prevailing wind directions at the 55 m height layer, based on sampling wind data. The prevailing wind direction is from the SE in January-February, shifts to NNW in March, and then to SSW from April to June. For the period from July to September, the prevailing direction is N. It is SSE in October, before returning to SE for November and December. A comprehensive analysis, in conjunction with Figure 1, reveals that the prevailing wind directions at the site are primarily aligned with several gaps on the periphery of the basin. Similarly, wind observation data from the GBT show that its prevailing wind direction is also oriented towards the site’s valley entrance [26].

**Figure 12:**
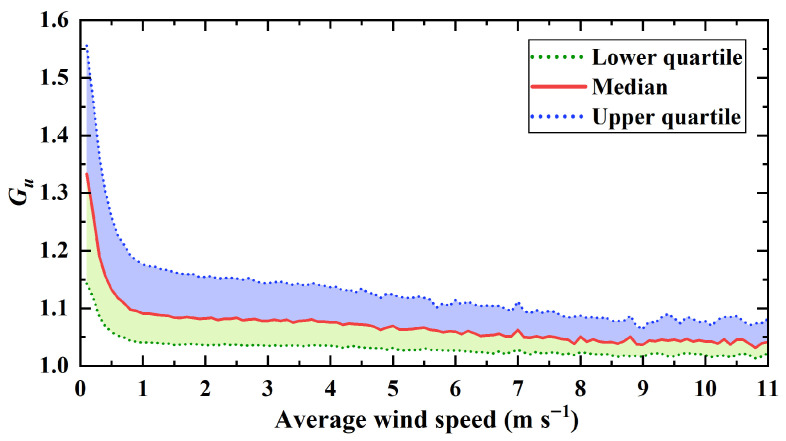
Variation in the gust factor with average wind speed.

To further investigate the turbulence characteristics of the wind field, the statistics of the gust factor ($G_{u}$) were analyzed. $G_{u}$ is defined as the ratio of the maximum wind speed to the average wind speed. Figure 12 illustrates the characteristics of $G_{u}$ under different average wind speed conditions, plotting the median, upper quartile, and lower quartile curves to reflect the variation trend and dispersion of the gust factor. As shown in the figure, $G_{u}$ exhibits a distinct non-linear decreasing trend as the average wind speed increases. When the average wind speed is less than 2 m/s, the gust factor decays rapidly with increasing wind speed, with the median dropping from over 1.33 to approximately 1.08. The relatively wide shaded area between the upper and lower quartiles during this phase indicates large data dispersion. This suggests that under low wind speed conditions, the airflow stability at the site is poor and is significantly influenced by local thermal turbulence or topographic disturbances, resulting in large fluctuations of instantaneous wind speed relative to the mean flow. Conversely, when the average wind speed exceeds 5 m/s, the curve gradually flattens, and the median stabilizes between 1.03 and 1.07. The narrowing interquartile range indicates that as the wind speed increases, the airflow becomes relatively stable, and the relative intensity of gust fluctuations diminishes.

### 3.3. Analysis of Daily Wind Environment Characteristics

**Figure 13:**
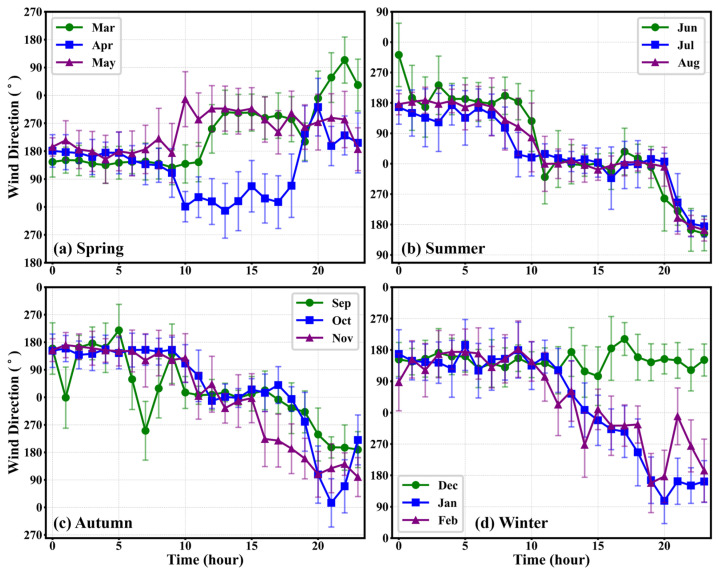
Daily wind direction distribution diagram.

**Figure 14:**
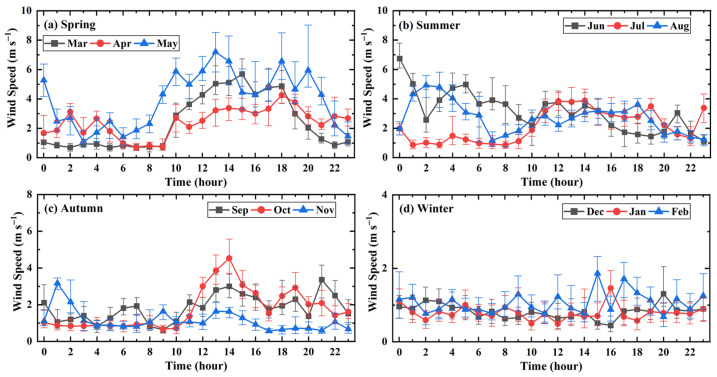
Daily wind speed distribution diagram.

To analyze the diurnal variation patterns, sampling wind data from the 1st, 11th, and 21st of each month were selected. The hourly wind speed data from these three days were aggregated to calculate statistics and plot the daily wind direction and speed distributions. If the proportion of anomalous entries in the selected daily wind data exceeded 10%, wind data from an adjacent, qualifying day were selected as the sample. “Daytime” was defined as the period from 08:00 to 20:00, and “nighttime” as the period from 20:00 to 08:00 of the following day. The resulting distributions for daily wind direction and wind speed are illustrated in Figure 13 and Figure 14, respectively.

In Figure 13, the solid lines represent the hourly mean wind direction, while the vertical error bars indicate the standard deviation of the wind direction angle within that hour. To accurately reflect the dispersion of circular wind direction data, the standard deviation was calculated using the Yamartino algorithm. The figure reveals a distinct diurnal reversal pattern in the wind direction. This pattern is analogous to local winds such as mountain-valley breezes or slope winds [27,28]. Generally, the prevailing wind direction during the nighttime is concentrated around 180° (southerly winds). In contrast, during the daytime, the wind direction shifts significantly, concentrating around 0° (northerly winds). This pattern is most regular and pronounced in summer and autumn. Particularly in summer, the relatively short error bars during the daytime indicate a stable northerly airflow. While spring and winter also exhibit this reversal trend, the data dispersion is larger, as evidenced by the longer error bars. For instance, in April (spring) and January (winter), the wind direction fluctuates intensely during the transition period.

The formation of this local wind system is significantly influenced by the topographical conditions of the Qitai site. The site is situated at the northern foothills of the eastern Tianshan Mountains, flanked by the snow-capped Tianshan Mountains to the south and by the oasis plain and the desert to the north [29]. This geographical setup creates a complete local circulation system. During the day, as the desert region heats up rapidly, warm air ascends towards the mountains, resulting in northerly winds at the site. Conversely, at night, cold, dense air from the mountains subsides, leading to southerly winds. Furthermore, the duration of these daytime northerly winds shows a high correlation with the seasons, a phenomenon influenced by the varying duration of solar radiation.

In Figure 14, the symbols connected by solid lines represent the median wind speed at that hour, while the upper and lower ends of the vertical error bars represent the upper and lower quartiles, respectively, reflecting the variation trend and dispersion of the data. As observed in the figure, the general trend for most months exhibits a distinct diurnal cycle, where daytime wind speeds are generally higher than those at night, with peak values typically occurring around 14:00. In addition, the greater the variation in wind speed, the more significantly the upper and lower quartile intervals increase. Spring exhibits the most intense diurnal variation. Notably, in May, wind speeds rise significantly after 08:00, with the median peak exceeding 7 m/s, and the large interquartile range suggests strong wind fluctuations during the day. Summer and Autumn show a similar trend of higher speeds during the day and lower at night, but the overall intensity is weaker than in spring. July and November show relatively low and flat diurnal variations, whereas June and August show a notable increase in nighttime wind speeds. Winter is the season with the lowest and most stable wind speeds throughout the year. The median wind speeds for December, January, and February remain largely below 2 m/s for most of the time, with minimal diurnal fluctuation and no obvious daytime increase in wind speed. This finding suggests that, from an operational standpoint for the radio telescope, high-frequency observations are better suited for nighttime conditions.

Unlike civil structures, where the primary concern is survival under extreme loads, the critical operational constraint for large radio telescopes is pointing accuracy under operational wind loads. For the QTT, the survival wind speed is designed to be 50 m/s. Given that the region averages only one gale day (>17.2 m/s, 10 m above ground level) per year, the risk of structural failure due to extreme winds is minimal. Instead, the primary research question focuses on the availability of observational windows for high-frequency science. Experience from the GBT indicates that wind speeds > 3 m/s begin to degrade pointing accuracy for observations above 40 GHz, while speeds > 5 m/s affect observations above 20 GHz [8]. Table 5 provides a statistical summary for 2021 based on sampling data from the 55 m height layer. It details the number of days on which wind speeds remained below specific thresholds (≤2, ≤4, ≤6, ≤8, and ≤10 m/s) for various proportions of the day. For this analysis, any day with more than 10% anomalous data was excluded, resulting in a total of 355 valid days. Therefore, based on the GBT’s operational experience, it can be inferred that the QTT will have well over 79 days per year suitable for observations above 40 GHz.

## 4. Conclusions

This paper investigates the wind environment characteristics at the QTT site based on data collected from a single wind tower throughout 2021. The main conclusions are as follows.

1. The generation of anomalous data from the wind tower is primarily attributed to rain and snow weather conditions conducive to freezing. Therefore, the selection of future wind observation equipment must place greater emphasis on anti-icing performance.
2. On an annual basis, the analysis of wind environment characteristics indicates that the magnitude of variation in wind speed and direction with height is minimal in the east–west direction but significant in the north–south direction, with winds predominantly originating from the north and south. For all southerly directions, α > 0.1. The wind distribution is strongly influenced by local terrain; as sensor height increases, the most frequent wind directions align more clearly with distant mountain gaps. The prevailing wind direction for the entire year is SSE, while the SSW is characterized by both high frequency of occurrence and high wind speeds. Overall, 90% of winds are below 4 m/s, with a maximum recorded wind speed of 22.29 m/s.
3. On a monthly basis, the analysis reveals that the distributions of wind speed and direction exhibit distinct seasonal cycles. The wind speed is relatively high in spring and summer, while it is relatively low in autumn and winter.
4. On a diurnal basis, the analysis shows a clear diurnal reversal pattern in wind direction, with predominantly northerly winds during the day and southerly winds at night. Nighttime wind speeds are generally stable and low, and this pattern is seasonally dependent, with the duration of calm conditions being longer in winter. Statistically, there were 79 days on which 90% of all winds were ≤2 m/s. Based on the operational experience of the GBT, it is inferred that the QTT will have more than 79 days per year suitable for high-frequency observations above 40 GHz.

This study provides foundational wind field data to support the antenna’s design for wind disturbance mitigation. It also lays the groundwork for more comprehensive site-wide wind field measurements and future wind field reconstruction efforts. Constraints in the sampling frequency of the existing wind monitoring system have hindered the analysis of the fluctuating wind component, and the continuity of data acquisition has been affected by icing events. To address this, a new wind observation system is currently being deployed at the QTT site, featuring a sampling rate upgraded to 1 Hz and sensors designed with anti-icing performance. For the missing measured data, machine learning will be used to restore them. Subsequent research will utilize this high-resolution data to deepen the characterization of the site’s wind field and inform the development of wind resistance strategies for the radio telescope.

## Figures and Tables

**Table 1:** Wind sensor technical specifications.

Meteorological Element	Measurement Range	Resolution	Precision
Wind direction	0~359°	1°	±3° (<12 m/s)
Wind speed	0~60 m/s	0.01 m/s	±0.2 m/s (<12 m/s)

**Table 2:** Anomalous data statistics.

Month	Number of Records	Number of Typical Anomalous Data	Heights of Typical Anomalous Data (m)	Time Interval of Typical Anomalous Data	Other Anomalies (Types; Number; Heights; Time)
January	44,640	0	None	None	None
February	40,320	830	15	25th 20~23 o’clock; 26th 0~12 o’clock	Markedly anomalous values; 3; 15 m; 22nd 6 o’clock
March	44,640	4708	All layers	15th 0~14 o’clock; 16~17th all day; 18th 0~16 o’clock	None
April	40,625	8	15, 55	22nd 7 o’clock; 24th 23 o’clock	Missing data; 2575; all layers; 7th 16 o’clock~9th 11 o’clock
May	44,640	0	None	None	None
June	43,200	0	None	None	None
July	44,640	0	None	None	None
August	44,640	1	25	17th 22 o’clock	None
September	43,200	0	None	None	None
October	44,640	112	25, 35, 45, 55	7th 10~11 o’clock	Markedly anomalous values; 6; 55 m; 7th 10 o’clock, 24th 9 o’clock
November	43,208	1922	All layers	4th 6~23 o’clock; 5th 0~10 o’clock	Time duplicates; 8; all layers; 17th 12~13 o’clock
December	44,640	0	None	None	None
Annual	523,033	7581	None	None	2592

**Table 3:** 16 standard wind directions.

Wind Direction	North	North-Northeast	Northeast	East-Northeast	East	East-Southeast	Southeast	South-Southeast
Symbol	N	NNE	NE	ENE	E	ESE	SE	SSE
Angle range (°)	348.76~11.25	11.26~33.75	33.76~56.25	56.26~78.75	78.76~101.25	101.26~123.75	123.76~146.25	146.26~168.75
**Wind** **Direction**	**South**	**South-Southwest**	**Southwest**	**West-Southwest**	**West**	**West-Northwest**	**Northwest**	**North-Northwest**
Symbol	S	SSW	SW	WSW	W	WNW	NW	NNW
Angle range (°)	168.76~191.25	191.26~213.75	213.76~236.25	236.26~258.75	258.76~281.25	281.26~303.75	303.76~326.25	326.26~348.75

**Table 4:** Monthly prevailing wind direction statistics.

Month	January	February	March	April	May	June
Prevailing wind direction	SE	SE	NNW	SSW	SSW	SSW
Proportion (%)	14.21	11.97	11.70	11.23	15.19	14.95
**Month**	**July**	**August**	**September**	**October**	**November**	**December**
Prevailing wind direction	N	N	N	SSE	SE	SE
Proportion (%)	12.48	11.98	11.38	13.21	14.63	17.15

**Table 5:** Statistics on the number of days with different wind speed thresholds accounting for different ratios.

Wind Speed (m/s)	60%	70%	80%	90%
≤2	245	162	117	79
≤4	342	328	300	230
≤6	353	346	335	316
≤8	355	354	347	334
≤10	355	355	354	345

## Data Availability

The data that support the findings of this study are available from the corresponding author (xuqian@xao.ac.cn) upon reasonable request.
